# A comparative study of the effects of three modes of 4-Nitroquinoline N-oxide induction of Sprague Dawley rats in animal models of oral leukoplakia

**DOI:** 10.1515/biol-2025-1263

**Published:** 2026-01-28

**Authors:** Mengyu Jiao, Peiyan Wang, Xiaofei Yu, Changqing Yuan, Pei Sun, Junjie Tong, Tianlu Wang, Jing Deng, Hui Zhang

**Affiliations:** Department of Stomatology, The Affiliated Hospital of Qingdao University, Qingdao 266003, China; School of Stomatology, Qingdao University, Qingdao 266023, China; Dental Digital Medicine & 3D Printing Engineering Laboratory of Qingdao, Qingdao 266003, Shandong, China

**Keywords:** 4-Nitroquinoline N-oxide, oral leukoplakia, animal modelling, p53 protein

## Abstract

Animal models are essential for advancing disease research. However, models for oral leukoplakia (OLK) remain relatively underexplored. This study sought to identify an optimal strategy for establishing a rat OLK model using 4-nitroquinoline 1-oxide (4NQO). The experimental groups included 0.002 % 4NQO drinking, 0.5 % 4NQO painting, and 0.002 % 4NQO drinking combined with 0.5 % 4NQO painting groups. Morphological changes, behavioral status, body weight, water intake and mortality were monitored. Histopathological features of rat tongue lesions were compared with those of human OLK. Drinking combined with painting group showed a large number and area of white lesions on tongue mucosa. The modeling effect of OLK in drinking group showed no significant difference from other groups, and the rats exhibited the best overall condition with no mortality. Painting group showed intermediate efficacy. Pathological manifestations in three groups were consistent with human OLK. Given its simplicity and superior safety profile, the 0.002 % 4NQO drinking protocol is recommended as the preferred approach for OLK model establishment.

## 1 Introduction

Oral leukoplakia (OLK) is a spectrum of oral mucosal disorders with an inherent risk of malignant transformation [1]. In the absence of timely and effective management, these lesions may progress to invasive cancer [2]. The risk of malignant transformation may be due to differences in the aetiology, lesion site, and follow-up duration [3], 4]. Among these, oral squamous cell carcinoma (OSCC) is the most frequent outcome. Globally, OSCC ranks as the sixth most common malignancy, with an overall 5-year survival rate of approximately 60 %, dropping to less than 28 % in advanced cases [5], 6]. Hence, early and effective intervention for OLK is of great significance in preventing malignant transformation and improving survival [7], 8]. Since the aetiology and pathogenesis of OLK remain incompletely understood, effectively curing OLK and reducing the risk of malignant transformation have always been scientific challenges.

Animal models have played an irreplaceable role in dynamic observations in OLK studies. Currently, few studies have been conducted on OLK animal models, most of which have focused on OSCC models [9], 10]. Previous reports have established Sprague Dawley (SD) rats as a preferred model for OLK induction [11], 12], achievable through exposure to 4-Nitroquinoline N-oxide (4NQO), 0.5 % benzopyrene/dimethylbenzanthracene mixed mutagenesis or cigarette smoke, typically delivered via drinking or painting [13], 14]. This study aimed to evaluate and compare different 4NQO-based induction strategies in SD rats – including drinking, painting, and a combination of both – to identify the most reliable and practical for OLK model establishment. Animal models can dynamically simulate the interactions between cellular, extracellular matrix, and signalling interactions within the microenvironment of OLK, providing an *in vivo* research platform for the occurrence, development, and treatment of OLK.

## 2 Materials and methods

### 2.1 Establishment of OLK rat model

Sixty-five four-week-old male SD rats (weighing 137.2 ± 5 g) were obtained from Jinan Pengyue Experimental Animal Breeding Co., Ltd. The rats were housed under standardized laboratory conditions in accordance with the National Standard for Laboratory Animal Environment and Facilities of China (GB 14925-2010/xg1-2011), with a 12 h light/dark cycle, relative humidity of 50 ± 15 %, and temperature maintained at 22 ± 2 °C.

Rats were randomly allocated into three experimental groups and one control group (*n* = 20 per experimental group; *n* = 5 for the control) [15], 16]. The experimental groups included 0.002 % 4NQO drinking, 0.5 % 4NQO painting, and 0.002 % 4NQO drinking combined with 0.5 % 4NQO painting groups.

Drinking group: 0.002 % 4NQO solution was prepared in drinking water, supplied *ad libitum* in alight-shielded bottles, and replaced twice weekly for 12 weeks [17].

Painting group: 0.5 % 4NQO solution was prepared in drinking water. The dorsum of the tongue mucosa was rubbed three times weekly, while the rats received normal drinking water for 12 weeks [18].

Drinking combined with painting group: Rats received both treatments. The dorsum of the tongue mucosa was rubbed with 0.5 % 4NQO three times weekly, and the rats were fed 0.002 % 4NQO for 12 weeks.

Oral mucosal changes, fur condition, overall condition, and body weight of the rats were monitored weekly. Photographs of the body and tongue lesions were taken at weeks 1, 8, and 12. Water intake was recorded daily. After 12 weeks, the rats were euthanised, and tongue specimens were harvested.

**Ethical approval:** The research related to animal use has been complied with all the relevant national regulations and institutional policies for the care and use of animals, and has been approved by the Laboratory Animal Welfare Ethics Committee of Qingdao University (batch number: 20200607SD13020201228025).

### 2.2 Haematoxylin and eosin (H & E) staining

Tongue lesions were excised, labeled, and fixed in 10 % neutral buffered formalin for 48 h. Paraffin sections were prepared and subjected to routine H & E.

### 2.3 Immunohistochemical analysis of p53

Paraffin-embedded sections were deparaffinised, rehydrated, and incubated with 3 % hydrogen peroxide for 20 min to quench endogenous peroxidase activity, followed by a 5 min rinse in phosphate-buffered saline. Antigen retrieval was performed by immersing slides in citrate buffer (pH 6.0), heating to boiling, maintaining at low heat for 15 min, cooling for 3 min, reheating for another 15 min, and allowing the slides to cool to room temperature. Sections were then incubated with anti-p53 primary antibody (1:1,000 dilution). After Diaminobenzidine staining, slides were briefly immersed in tap water preheated to 75–80 °C for 10–15 s, rinsed for 5 min, dehydrated, and mounted. Staining results were observed and imaged under an inverted light microscope.

### 2.4 Histopathological assessment of human OLK

To verify the validity of the rat OLK model, H & E-stained slices from patients clinically diagnosed with OLK at the Department of Oral Mucosa Diseases, Affiliated Hospital of Qingdao University were reviewed. Specimens were categorized into mild–moderate or moderate–severe epithelial dysplasia, and histopathological similarities between human and rat lesions were evaluated. This study was approved by all patients and the Ethics Committee of the Affiliated Hospital of Qingdao University (ethics approval number: QYFYWZLL29865).

### 2.5 Statistical analysis

GraphPad PRISM 8.0 software was used for statistical analyses. All experimental data are expressed as mean ± standard deviation (*X̄* ± SD). One-way ANOVA was used for comparisons among groups; *t*-test was used for comparison between two groups, and approximate *F*-test was used for those with uneven variances. Statistical significance was defined as *P* < 0.05, with **P < 0.01 and ***P < 0.001 indicating highly significant differences.

## 3 Results and discussions

### 3.1 Morphological changes and general condition

**Figure 1:**
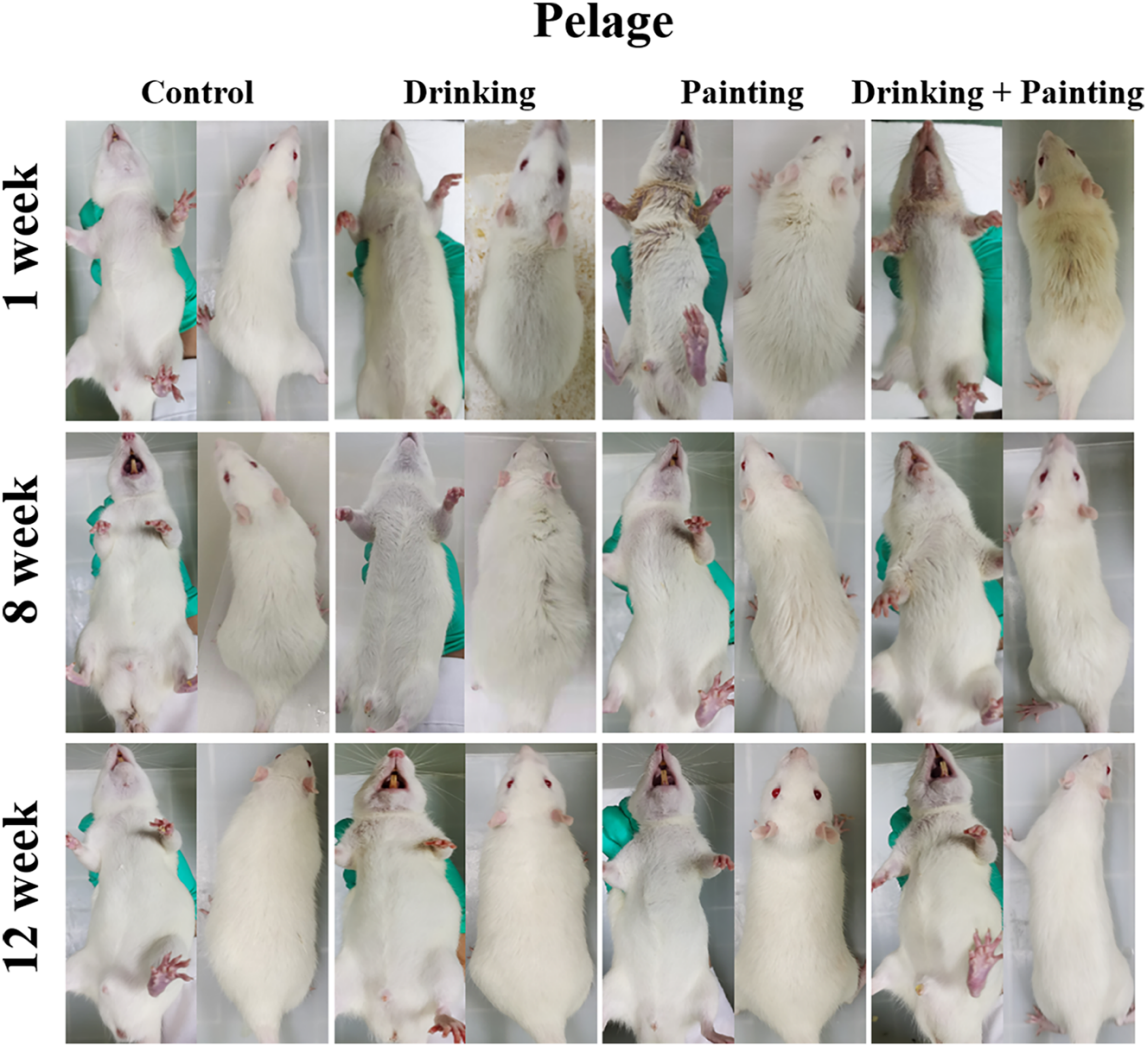
Changes in fur among rats in all groups during the modeling period.

The morphological changes and general condition of the rats are summarized in Figure 1.

Control group: Rats maintained smooth, glossy fur without hair loss and showed consistently normal behavior.

Drinking group: Rats maintained shiny fur, normal activity, and good overall condition throughout the 12-week period, with only occasional loose stools observed at week 12.

Painting group: Rats maintained dull, dry fur with partial alopecia in the mandibular region after one week, accompanied by poor overall behavior despite normal mobility. Mandibular fur returned to normal by week 8, although dorsal fur remained dry; both activity and mental status improved. By week 12, fur condition, defecation, movement, and overall behavior had normalized.

Drinking combined with painting group: Rats initially demonstrated complete mandibular alopecia, moist marginal fur, and yellowish, dry dorsal fur, along with diminished mental responsiveness. Mandibular fur recovered by week 8, although dorsal fur remained dull, and their mental status improved. By week 12, fur texture and behavior were normal, but loose stools were again noted.

At the beginning of the experiment, the external appearance of the rats was significantly different among the three modelling methods. The drinking group did not differ from those in the control group. However, in the painting and drinking combined with painting groups, the oral mucosa was damaged owing to direct stimulation by high concentrations of 4NQO, resulting in increased salivation and hair loss in the mandibular area after long-term immersion in saliva. These conditions were more severe in the drinking combined with painting group, likely exacerbated by repeated handling during thrice-weekly applications, which may have induced stress-related fur deterioration. These conditions gradually disappeared as the experiment progressed, with both fur condition and behavior returning to normal. Notably, rats in the painting and drinking combined with painting groups were subjected to stimulation with high concentrations of 4NQO and daily application of the carcinogen. By week 12, loose stools were noted in drinking and drinking combined with painting groups, which may have been due to gastrointestinal irritation caused by the prolonged systemic exposure to 4NQO. However, this phenomenon was not observed in the painting group.

### 3.2 Morphological changes of tongue mucosa

**Figure 2:**
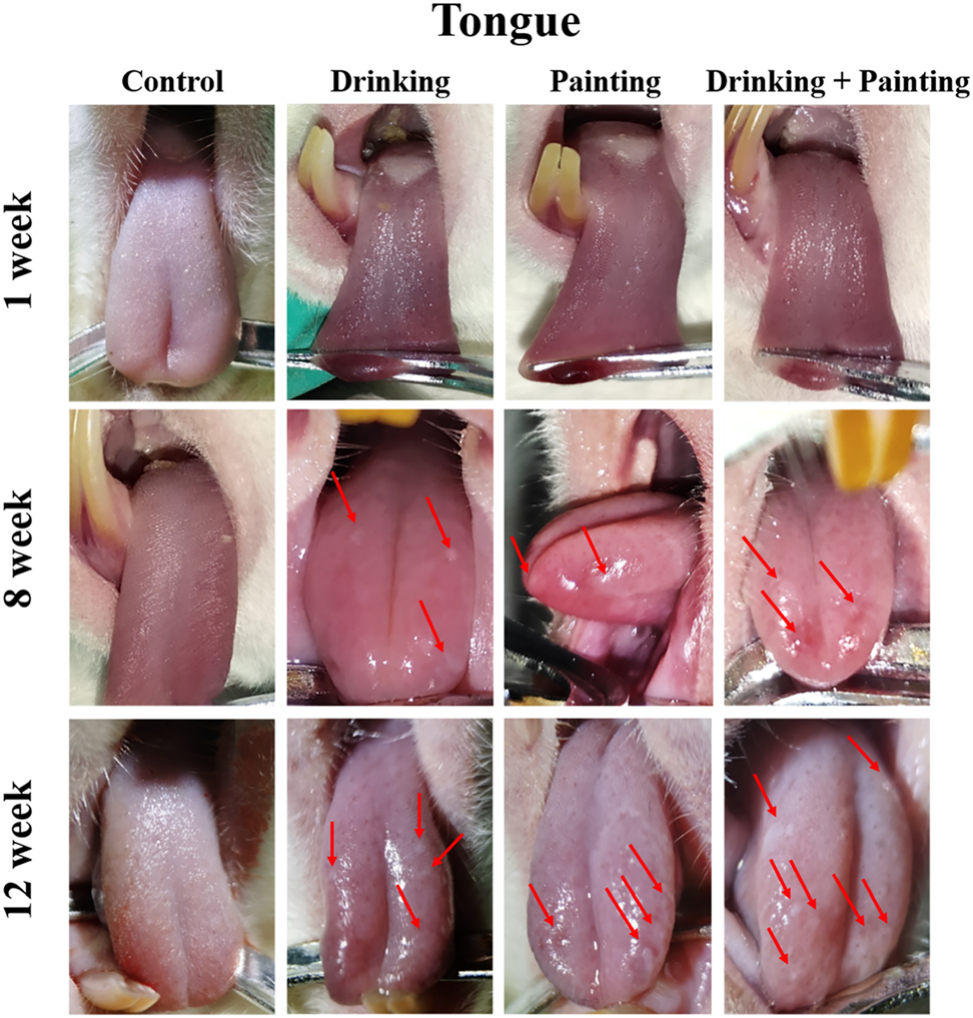
Morphological changes of tongue mucosa among rats in all groups during the modeling period.

As shown in Figure 2:

Control group: Throughout the entire experiment period, tongue mucosa remained uniformly pink with no signs congestion or erosion.

Drinking group: The tongue mucosa appeared normal after one week. By week 8, small white lesions emerged on the dorsal surface in some rats and increased considerably over time. By week 12, three to four homogeneous white lesions were distributed across dorsum of the tongue. The lesions enlarged to approximately 1.0 × 1.5 mm^2^ and exhibited a raised appearance on the mucosal surface.

Painting group: The tongue mucosa appeared normal after one week. By week 8, small white lesions accompanied by mild hyperaemia and erosion appeared on the tongue tip and margins, and enlarged progressively. By week 12, three to four homogeneous white lesions were present on the tongue tip and margins, accompanied by mild hyperaemia and erosion. Lesions increased to approximately 1.5 × 1.5 mm^2^ and were partially protruded from the surface of the mucosa.

Drinking combined with painting group: The tongue mucosa appeared normal after one week. By week 8, small white lesions with mild hyperaemia and erosion appeared on the tongue tip and dorsum and increased substantially over time. By week 12, five to seven homogeneous white lesions were scattered on the dorsum and margins of the tongue, accompanied by mild hyperaemia and erosion. Lesions increased markedly, ranging from approximately 1.0 × 1.0 mm^2^ for smaller lesions to 2.0 × 3.0 mm^2^ for larger ones, and were partially raised above the surface.

The rats in the three experimental groups appeared white lesions in different areas of the tongue mucosa by week 8. The lesions were predominantly located on dorsal tongue in the drinking group, likely because the drinking posture primarily exposes the dorsal mucosa to 4NQO [19]. The painting method used in previous studies required animals to be repeatedly anaesthetised and painted on the same area, leading to a high risk of death due to intestinal obstruction. In this study, we modified the painting method to avoid anesthesia by gently opening the rat’s mouth and swabbing the anterior two-thirds of the tongue with a 4NQO-soaked cotton swab. Consequently, lesions in the painting group were mainly concentrated on the tongue tip and margins. In the drinking combined with painting group, the lesions were concentrated on the dorsum, tip, and margins of the tongue. By week 12, lesions in all three experimental groups increased in both size and number, with the highest number of lesions noted in the drinking combined with painting group. Mild erosion was observed in some lesions in the painting and drinking combined with painting groups, which was related to the repeated use of 4NQO.

### 3.3 Drinking condition

**Figure 3:**
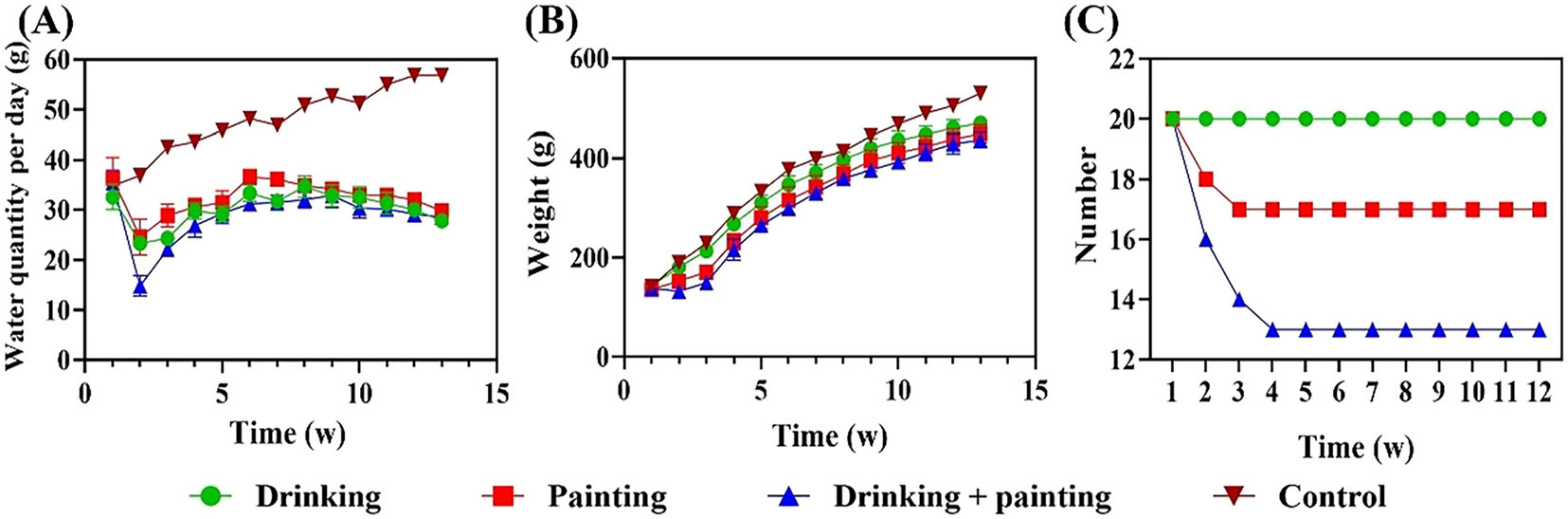
Changes in water intake, weight and mortality in all groups during the modeling period. (A) Water intake, (B) weight, (C) mortality.

As shown in Figure 3A, the water intake of the control group gradually increased with weight and age. In the early stages of the experiment, water intake decreased sharply in all three experimental groups. In the drinking group, this was primarily because the rats did not prefer 4NQO. In the painting group, the decrease may be attributed to mucosal irritation caused by swabbing the oral cavity with 4NQO- Cotton swab. In the drinking combined with painting group, both factors were present. Later, water intake gradually increased owing to the need for growth, taste adaptation, and local stimulation. During the later stage of the experiment, water intake in the three experimental groups gradually decreased and formed an inverted ‘S-shaped’ pattern by week 12. The overall trends were similar among the drinking, painting, and drinking combined with painting groups, with no statistically significant differences observed between them. However, all experimental groups exhibited significantly lower water intake than the control group (*P* < 0.0001). The observed intake patterns reflect a transition from initial aversion to partial adaptation to 4NQO exposure. Additionally, long-term use of 4NQO may gradually impair systemic physiological functions, resulting in decreased water intake during the late phase of the experiment.

### 3.4 Weight condition

As shown in Figure 3B, the weight gain trend of the drinking group was similar to that of the control group; however, the weight was significantly different from that of the control group during the corresponding period (*P* < 0.05). By week 1, no significant weight changes were observed in the painting group, whereas a downward trend was observed in the drinking combined with painting group. From week 2 onward, rats in the painting and drinking combined with painting groups began to slowly gain weight, which was significantly different from that of the control group during the corresponding period (*P* < 0.01). No significant differences in weight were observed among the drinking, painting, and drinking combined with painting groups at any time point. Overall, all three experimental groups exhibited lower weights compared with the control group, particularly in the early stages of the experiment, which indicated that 4NQO exposure exerted substantial effects on the general health and physiological status of the rats.

### 3.5 Mortality rate

As shown in Figure 3C, no deaths occurred in the drinking group throughout the experiment. In contrast, three rats in the painting group and six rats in the drinking combined with painting group died. The deaths occurred at the beginning of the experiment. After week 4, none of the rats in the three experimental groups died. A significant difference in mortality was observed among the three groups (*P* < 0.0001), with the drinking group exhibiting the lowest mortality rate.

### 3.6 Histopathological analysis

**Figure 4:**
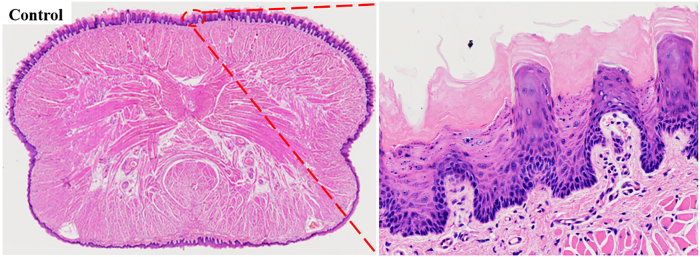
H & E staining images (200×) of tongue section panoramic and normal mucosal epithelium in control group.

**Figure 5:**
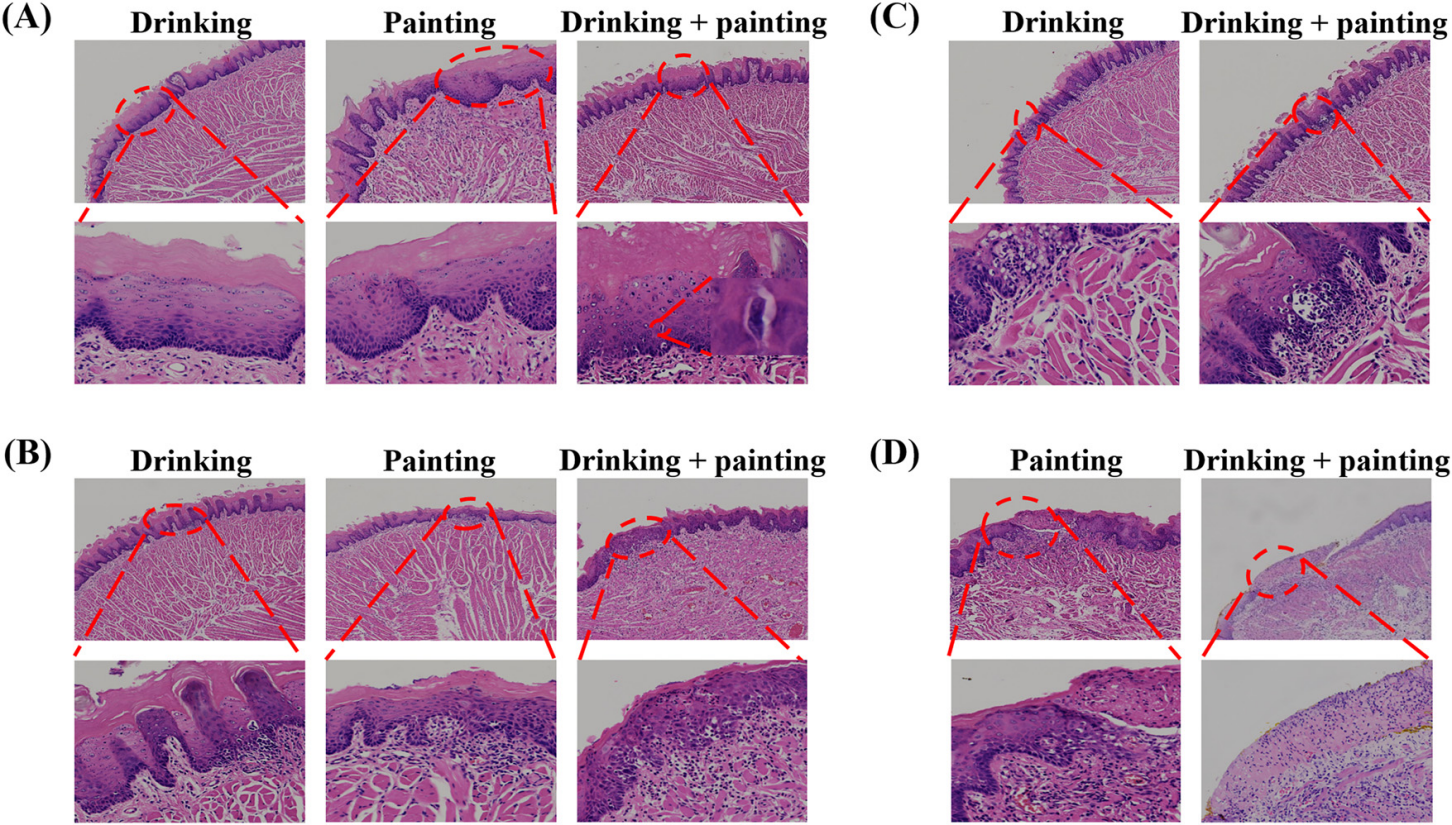
H & E staining images (200×): (A) Pathological manifestations observed in all three experimental groups. (B) Pathological manifestations observed in all three experimental groups. (C) Pathological manifestations observed in both drinking group and drinking combined with painting group. (D) Pathological manifestations observed in both painting group and drinking combined with painting group.

In the control group, the dorsal tongue mucosal epithelium displayed clear stratification, with tightly arranged keratinized layer cells, a single row of uniformly aligned short columnar basal cells, an intact basement membrane, and moderate nuclear size and epithelial peg length. No evidence of epithelial dysplasia was observed, consistent with normal mucosal morphology (Figure 4). By week 12, pathological manifestations were observed in all three experimental groups, including epithelial hyperplasia, excessive keratinisation of the keratin layer, thickening and shortening of epithelial pegs, increased basal cell layers, spinous layer thickening, granulation tissue proliferation, and abnormal transparent keratinisation granules, with abnormal nuclear division observed in the drinking combined with painting group (Figure 5A). Additionally, epithelial layer disorder, basal cell polarity loss, cell polymorphisms, and lymphocyte infiltration were observed (Figure 5B). Basal cell vacuolar changes and basement membrane degradation were observed in the drinking and drinking combined with painting groups (Figure 5C). Pathological manifestations including epithelial layer destruction and inflammatory granulation hyperplasia were observed in the painting and drinking combined with painting groups (Figure 5D).

At 12 weeks, all experimental groups showed abnormal epithelial hyperplasia, epithelial layer disorder, and basal cell polarity loss, confirming successful induction of OLK in the rat model. Abnormal nuclear division in the drinking combined with painting group suggested a tendency towards OSCC, indicating that the progression in this group from normal squamous epithelium to dysplastic squamous epithelium was faster than that in the other two groups. Continued 4NQO exposure would likely accelerate malignant conversion in the drinking combined with painting group. Basal cell liquefaction, degeneration, and vacuolar changes observed in the drinking and drinking combined with painting groups resembled pathological features of oral lichen planus (OLP). Because no animal model for OLP currently exists, these findings may offer insight into future model development. Epidermal destruction and inflammatory granulation tissue hyperplasia observed in the painting and drinking combined with painting groups were consistent with the diagnosis of oral mucosal ulcers. This may be related to the direct rubbing stimulation of 4NQO carcinogens during the two modelling processes. Overall, histopathological results indicate that all three modelling methods successfully induced OLK within 12 weeks, and the severe pathological progression was faster in the drinking combined with painting group than in the other two groups.

### 3.7 Expression of p53

**Figure 6:**
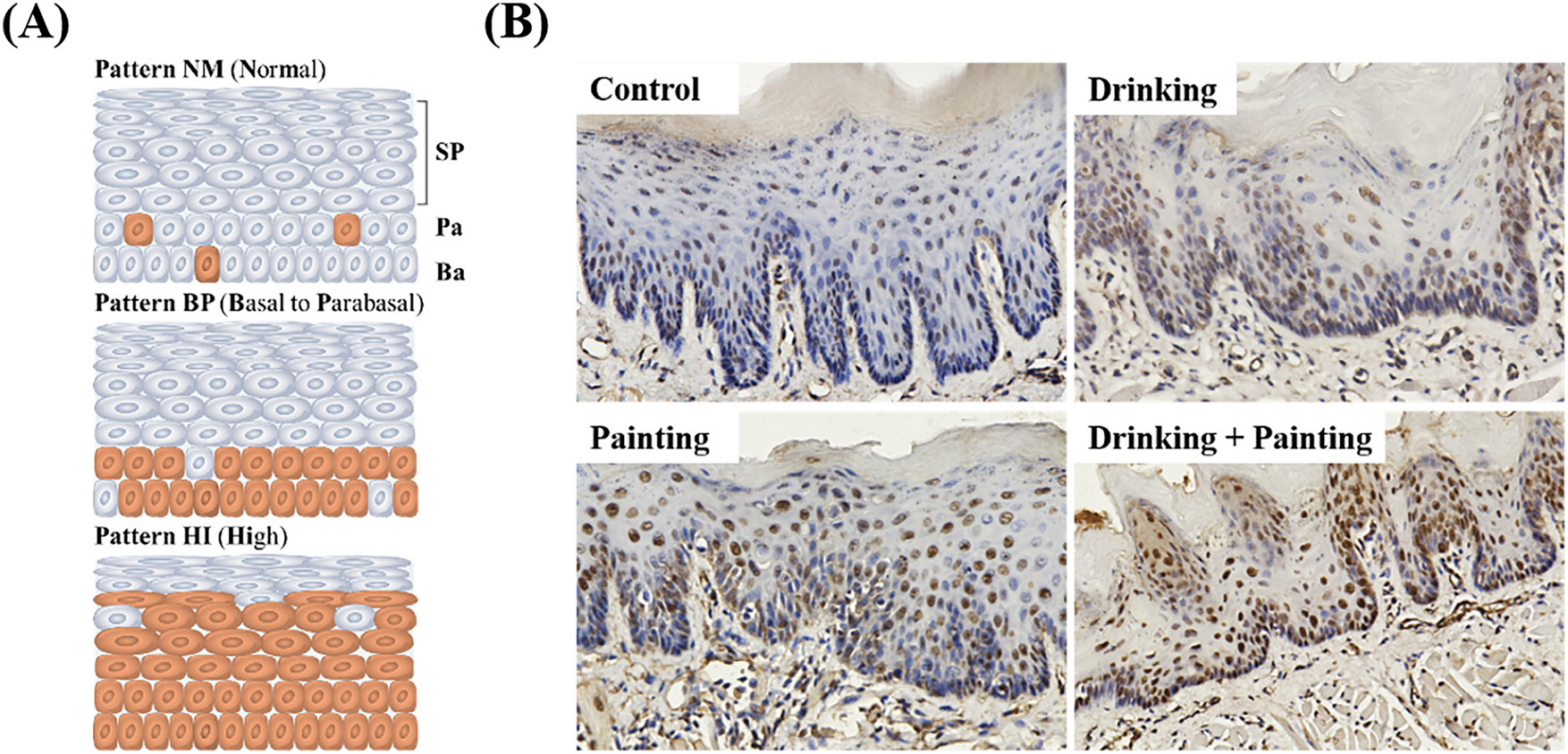
Expression of p53: (A) Schematic diagram of p53 immunohistochemical staining degree (B) immunohistochemical images of p53 in rats (200×).

p53 profoundly affects the biological behaviour of OLK by integrating cellular metabolic reprogramming and epigenetic regulatory networks. Its expression is widely used as an immunohistochemical diagnostic criterion for abnormal epithelial hyperplasia, and a diagnostic model based on p53 staining patterns has been established [20]. As shown in Figure 6A, the NM staining pattern is characterized by weak p53 positivity confined to the basal layer, with complete negativity in suprabasal cells, consistent with normal mucosal epithelium. In the BP staining pattern, p53 positive cells are restricted to the basal layer, with weak to moderate staining intensity, which is more common in mild epithelial dysplasia. In the HI staining pattern, p53 positive cells expanded horizontally from the basal layer to nearly the entire epithelium, which is characteristic of moderate-to-severe dysplasia. Therefore, tongue specimens from each group were analysed using immunohistochemistry for p53. As shown in Figure 6B, p53 positive cells in the control group were scattered in the basal layer, and the positivity rate was very low, corresponding to the NM staining pattern. The p53 positive cells in the three experimental groups were gathered within dysplastic epithelial regions and distributed across the basal, parabasal, and spinous layers, with markedly elevated positivity consistent with the HI staining patterns. These results confirmed that all three modelling methods could induce epithelial dysplasia and successfully establish rat OLK models.

### 3.8 Comparison of pathological manifestations of OLK in animal models and humans

**Figure 7:**
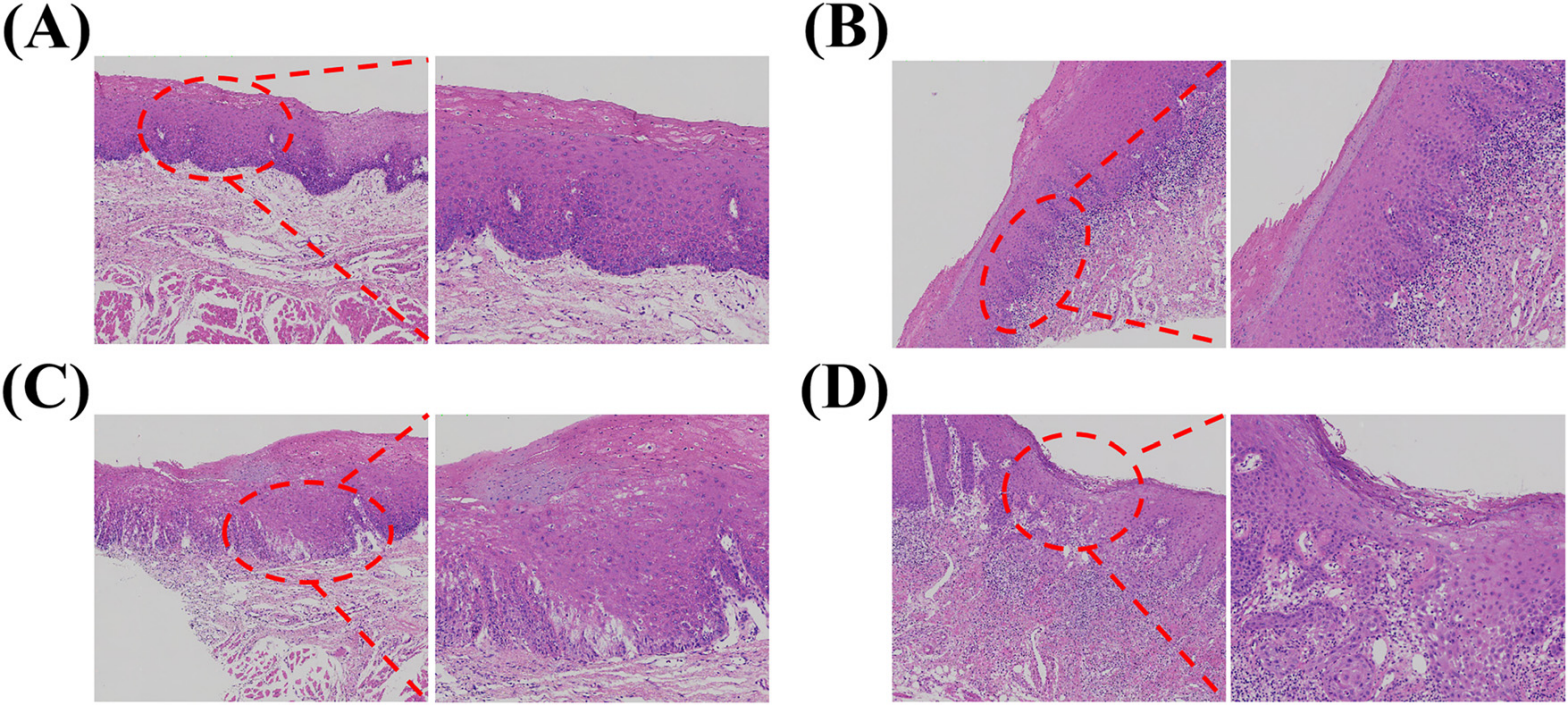
Human H & E staining images (10×): (A) Epithelial pegs thickening and shortening, (B) basal cell polarity loss, (C) basal cell vacuolar change, (D) upper cortex destruction.

Figure 7A shows the pathological manifestations of human OLK with mild-to-moderate dysplastic, characterized by epithelial hyperplasia, hyperkeratinisation of the keratotic layer, and thickening and shortening of the epithelial pegs – features highly similar to those shown in Figure 5A. Figure 7B shows the pathological manifestation of moderate-to-severe dysplastic leucoplakia in humans, in which the epithelial level is disturbed and the basal cells lose polarity. This is similar to the results shown in Figure 5B. The pathological manifestations shown in Figure 7A and B were observed in all three experimental groups, further demonstrating the reliability of the rat model. Figure 7C shows human mild to moderate dysplasia with basal cell vacuolar degeneration, closely resembling the features in Figure 5C. These manifestations were found in both the drinking and drinking combined with painting groups, indicating that the lingual mucosal epithelial cells in these two groups had metabolic abnormalities or mild damage. Both methods can simulate the pathological manifestations of mild-to-moderate OLK. Figure 7D shows human moderate-to-severe dysplasia with epithelial destruction, consistent with Figure 5D, which was observed in both the painting and drinking combined with painting groups. It is characterised by severe lesions and an increased risk of cancer. To a certain extent, the similarity between animal models and human lesions indicates that all three modelling methods can simulate pathological changes of human OLK, reinforcing their utility for mechanistic studies and preclinical drug development.

Experimental animal models offer several advantages, including short research cycles, high reproducibility, low risk, and controllable experimental conditions. An ideal animal model should resemble the corresponding human disease as closely as possible in anatomical and physiological characteristics and histopathological features [21]. A higher degree of similarity indicates higher research accuracy; therefore, establishing animal models is crucial. Many SD rats are derived from inbred strains with highly uniform genetic backgrounds, thereby reducing inter-individual variability. As a widely used experimental species, SD rats have been extensively characterized at the genomic level, providing valuable reference data for genetic and pathological studies [22]. Their physiological and metabolic systems also closely resemble those of humans. Moreover, compared with mice, the larger body size of SD rats allows for the collection of more tissue from tongue lesions, facilitating downstream analyses. For these reasons, SD rats were selected for the present study. OLK is pathologically characterized by epithelial dysplasia, and preventing or reversing dysplastic progression is of great significance for inhibiting malignant transformation [23]. Therefore, establishing an OLK animal model is essential for elucidating disease initiation, carcinogenic evolution, and immune microenvironment regulation. Masuda reported that 4NQO-induced rat tongue carcinogenesis proceeds through a stepwise process from normal epithelium to OLK and ultimately to OSCC, providing a framework for early OSCC detection [24]. Similarly, Sari observed that epithelial atypia appeared in lesion sites prior to carcinoma development in a 4NQO-induced SD rat model, consistent with OLK pathology [25]. Although 4NQO is widely used to induce oral cancer models, studies focusing specifically on 4NQO-induced OLK rat models remain limited. Both the 0.002 % 4NQO drinking and 0.5 % 4NQO painting methods can induce OSCC and OLK in animal models, providing the rationale for dosage selection in our study. However, the painting process is complex and requires repeated anaesthesia, resulting in high mortality in rats. In comparison, the drinking method is simpler to operate, minimally disruptive to daily activity, and highly effective for model induction. Zigmundo reviewed prior studies and noted that although both drinking and painting methods could successfully induce oral carcinogenesis, most researchers preferred the drinking approach due to its ease of use and safety [26]. The modeling strategies recommended in our study are consistent with this consensus. In this study, we compared the 0.5 % 4NQO painting method with the 0.002 % 4NQO drinking method and innovatively combined the two methods to establish a faster, safer, and more effective OLK animal model.

## 4 Conclusions

In conclusion, the OLK rat model was successfully established within 12 weeks using three different 4NQO induction methods. All models exhibited pathological manifestations highly consistent with human OLK. Comprehensive comparison weight, general condition, water intake, mortality, tongue morphological and histopathological changes, and p53 immunohistochemistry revealed that the 0.002 % 4NQO drinking method represented the safest and most effective approach for OLK induction.
